# Tissue loading created during spinal manipulation in comparison to loading created by passive spinal movements

**DOI:** 10.1038/srep38107

**Published:** 2016-12-01

**Authors:** Martha Funabashi, Gregory N. Kawchuk, Albert H. Vette, Peter Goldsmith, Narasimha Prasad

**Affiliations:** 1 Department of Physical Therapy, University of Alberta, Edmonton, AB, Canada; 2 Department of Mechanical Engineering, University of Alberta, Edmonton, AB, Canada; 3 Glenrose Rehabilitation Hospital, Alberta Health Services, Edmonton, AB, Canada; 4 Department of Mechanical Engineering, University of Calgary, Calgary, AB, Canada; 5 Department of Mathematical and Statistical Sciences, University of Alberta, Edmonton, AB, Canada

## Abstract

Spinal manipulative therapy (SMT) creates health benefits for some while for others, no benefit or even adverse events. Understanding these differential responses is important to optimize patient care and safety. Toward this, characterizing how loads created by SMT relate to those created by typical motions is fundamental. Using robotic testing, it is now possible to make these comparisons to determine if SMT generates unique loading scenarios. In 12 porcine cadavers, SMT and passive motions were applied to the L3/L4 segment and the resulting kinematics tracked. The L3/L4 segment was removed, mounted in a parallel robot and kinematics of SMT and passive movements replayed robotically. The resulting forces experienced by L3/L4 were collected. Overall, SMT created both significantly greater and smaller loads compared to passive motions, with SMT generating greater anterioposterior peak force (the direction of force application) compared to all passive motions. In some comparisons, SMT did not create significantly different loads in the intact specimen, but did so in specific spinal tissues. Despite methodological differences between studies, SMT forces and loading rates fell below published injury values. Future studies are warranted to understand if loading scenarios unique to SMT confer its differential therapeutic effects.

Spinal manipulative therapy (SMT) is a clinical intervention for low back pain which, by some estimates, is the most frequently used form of complementary and alternative medicine (CAM)^1^. Similarly, SMT is also one of the most studied CAM interventions with over 250 systematic reviews and 550 randomized controlled trials since 2000.

Unfortunately, this volume of literature is polarized, with an almost equal number of publications describing the benefit of SMT or its lack of therapeutic effect. Toward understanding this discrepancy, recent studies have suggested that some forms of low back pain respond to SMT while others do not. Specifically, SMT creates significant changes in spinal stiffness, muscle response and disc diffusion in SMT responders while these same changes are not observed in SMT non-responders^2^,^3^,^4^. In addition, approximately 30–50% of persons who receive SMT experience some kind of benign adverse event that typically self-resolves in 24–48 hours^5^,^6^,^7^, with a minority of patients experiencing serious adverse events^8^,^9^,^10^. However, causation in these cases remains controversial^7^,^9^,^10^,^11^,^12^.

To better understand this range of therapeutic responses in persons receiving SMT, it is important to understand the loads that are imparted to the spine as a result of SMT application. In response, we have developed a robotic technique that is capable of quantifying the *in-situ* loads experienced by spinal segments as well as specific spinal tissues. By robotically replicating the same kinematics experienced during SMT and sequentially removing specific spinal tissues between kinematic replication, the change in loading following spinal tissue removal can be considered to be the load experienced by that specific tissue. Using this approach, we have shown that, during the application of SMT on the skin overlying the L3 transverse process, the intervertebral disc is the spinal structure that experiences the greatest loads^13^.

While this approach can determine the load distribution within spinal tissues affected by SMT, we do not yet understand the importance of the magnitude of these loads. Specifically, we lack a frame of reference to understand whether these loads are larger, smaller or equal to those experienced by the spine during everyday motions. Having this comparison is fundamental toward identifying if unique spinal loads arise from SMT. If unique SMT loads exist compared to those arising in typical spinal motion, then it may be these loads that confer the beneficial (or harmful) effects of SMT. While previous investigations have reported vertebral motion, intradiscal pressure and spinal loading as a result of SMT^13^,^14^,^15^ or typical spine movements^16^,^17^, methodological differences between these investigations preclude the direct comparison of biomechanical characteristics (e.g., spinal loading) during SMT application and typical movements. Therefore, a unified investigation of spinal loading during the application of both SMT and typical movements in one study within a single model using a standardized testing protocol is needed to identify if SMT provides unique loading features.

Given the above, the objective of this study was to compare loads in a lumbar spine segment arising from SMT and passive spinal movements (flexion, extension and axial rotation) using an established cadaveric model. We hypothesized the existence of significantly different forces arising from SMT when compared to those arising from passive movements.

## Methods

### Sample Size Calculation

A sample size calculation was conducted based on the data previously reported by Kawchuk and colleagues (2010)^13^ using the General Power Analysis Program (G*Power 2) (University of Trier, Germany). With statistical power set to 80%, a two-tailed test having a level of significance at 0.05 (5%) and an effect size of 0.99–1.2, a sample size of 9 was calculated. Five additional specimens were included to account for possible specimen loss due to testing complications, resulting in 14 cadaveric porcine specimens tested. All experimental protocols of this study were approved by the Animal Care and Use Committee of the University of Alberta.

### Specimen Preparation

**Figure 1:**
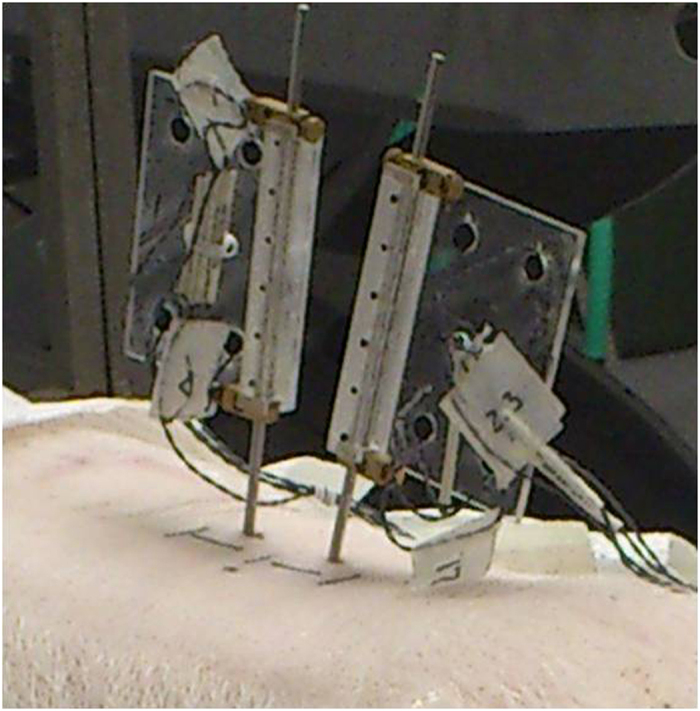
Rectangular flags with 4 infrared light-emitting diode markers attached to bone pins drilled into L3 and L4 vertebrae.

Fourteen fresh porcine cadavers (Duroc X [Large White X Landrace breeds]) of approximately 60–65 kg were included in this study. In each intact cadaver, ultra-sound imaging and needle probing were used to identify the L3 and L4 vertebrae and the L3/L4 left facet joint (FJ). Bone pins were drilled into the L3 and L4 vertebral bodies and a rectangular flag having 4 infrared light-emitting diode markers was attached to the upper end of each bone pin (Fig. 1).

Each cadaveric pig was then positioned in a neutral, prone position on a hinged hardwood board that was fixed to a flexion-extension table (Leander Health Technologies, Lawrence, KS). The L3 and L4 vertebrae were positioned above and below the hinge line, respectively, and the cadaver fixed to the table by a strap around the lower thoracic region^18^. SMT was applied with the cadaver in this orientation. To apply passive movements, the portion of the cadaver superior to the hinge (L3) was kept stationary while the portion of the cadaver located inferior to the hinge (L4) was moved. Following the application of SMT and passive lumbar movements on the intact porcine cadaver (detailed in the following sections), the lumbar spine was removed *en bloc*^13^. The L3/L4 spinal segment was cleaned of non-ligamentous tissues, sealed in a plastic bag and kept refrigerated at 3 °C for less than 5 hours until potting and testing on the following day^19^. The specimen was kept moist with physiologic saline throughout preparation, embedding and testing^20^,^21^. Due to complications in robotic calibration for attaining neutral position alignment, two specimens were excluded and, therefore, data from 12 specimens were analyzed. Given the fragile nature of the intertransverse ligaments and their frequent damage during *en bloc* spinal removal, all specimens had their intertransverse ligaments removed prior to testing. All methods in this study were performed in accordance with the NIH Guide for the Care and Use of Laboratory Animals.

### Application of SMT

A trained clinician with 3 years of clinical experience was instructed to apply SMT using a “hypothenar push” manipulation in which the pisiform bone of the clinician’s hand was positioned on the skin of the specimen overlying the left L3/L4 FJ^22^. To measure SMT force-time characteristics, a pressure array (Pressure Profile System, Inc. Los Angeles, CA) was placed between the clinician’s hands and the cadaver skin. The pressure array was composed of 10 × 10 pressure sensors with a pressure sensitivity of 0.15% and a recording rate of 120 Hz.

### Application of passive motion

**Figure 2:**
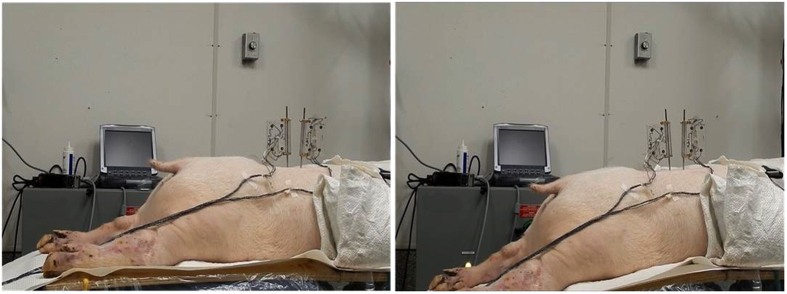
Passive flexion movement performed by the automatic flexion movement of the flexion-extension table.

**Figure 3:**
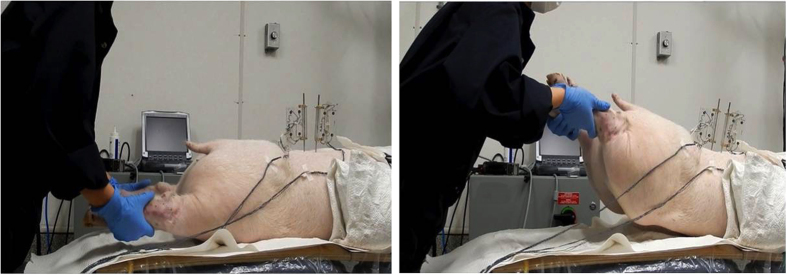
Passive extension movement performed by manually moving both lower limbs in the upward direction.

**Figure 4:**
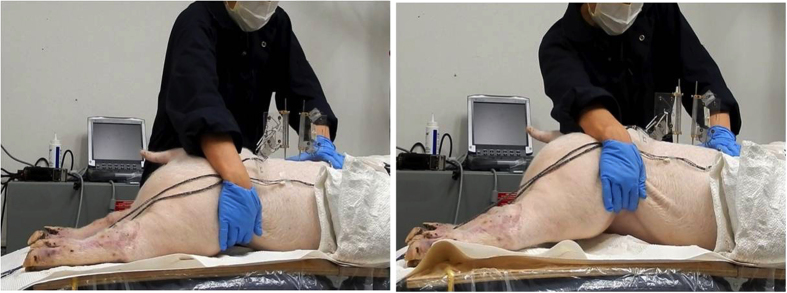
Passive left axial rotation movement performed by manually stabilizing L3 and manually rotating the pelvis to the left side.

Three passive lumbar movements were performed: flexion, extension and left axial rotation. Passive flexion movement was performed by the automatic flexion movement of the flexion-extension table where the table was moved mechanically to a maximum of 20° of flexion at an approximate rate of 0.8°/s (Fig. 2). Passive extension was performed by manually moving both lower limbs in an upward direction at an average rate of 5°/s until the maximum extension between the L3/L4 segment was reached and the L2/L3 segment started to move visually (Fig. 3). Passive left axial rotation was performed by manually stabilizing L3 and manually rotating the pelvis to the left side at an average rate of 3°/s until the maximum range of rotation was reached and the L3 bone pin began to rotate visually (Fig. 4). Each passive lumbar movement was performed twice, and the data from the second movement was used for analysis.

### Segmental motion recording

During the application of SMT and passive lumbar movement, the resulting motions of the L3 and L4 vertebrae were recorded by an optical tracking system (Optotrak Certus, NDI, Waterloo, Canada) in 3 dimensions at a rate of 400 Hz with a 0.01 mm system resolution and a 0.15 mm rigid body resolution.

### Robotic Testing

After application of SMT and passive motions, the L3/L4 motion segment was removed as described previously, and the specimen potted in a vertical orientation using dental stone (Modern Materials, South Bend, IN) with the intervertebral disc positioned parallel to the horizontal plane as aligned by a projected laser beam. The caudal end (L4) of the potted spinal segment was fixed to a 6-axis load cell (AMTI MC3A-1000, Advanced Mechanical Technology, Inc., Watertown, MA), which was mounted rigidly to a parallel robot platform (Parallel Robotics Systems corp., Hampton, NH), such that the anatomical axes of the specimen aligned with both the load cell axes and the robot axes as follows: x = mediolateral, y = anterioposterior, and z = superioinferior.

To calibrate the system, a series of known translations and rotations were provided to the robot and the resulting change in position of the optical markers on L4 recorded. This calibration provided the position and orientation of the L4 marker set with respect to the robotic platform.

**Figure 5:**
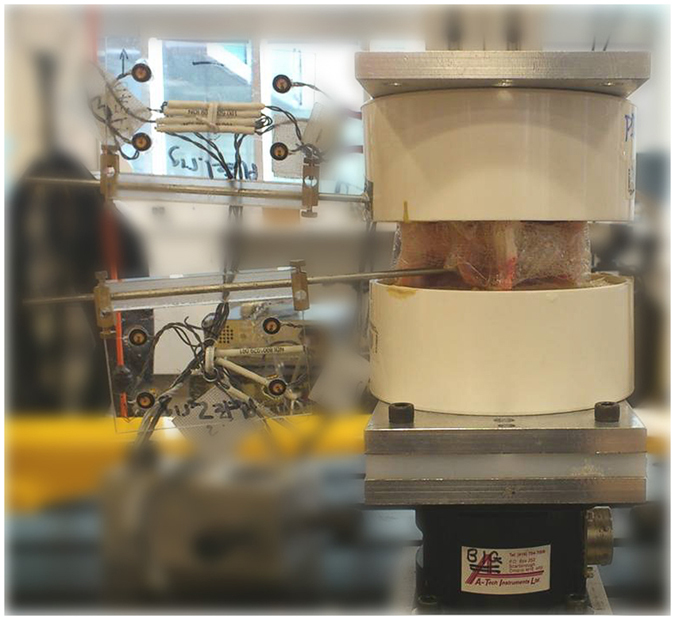
Potted spinal segment with L4 mounted to the 6-axis load cell and L3 fixed to a stationary cross beam.

The cranial end of the potted specimen was then fixed to a stationary cross beam and the segment positioned in the same position and orientation recorded previously from the cadaver’s intact neutral pose (Fig. 5). By following the procedures described by Goldsmith *et al*.^23^, the marker movements caused by SMT and each of the 3 passive motion were then transformed into robot trajectories that replicated the relative motions between L3 and L4 vertebrae recorded by the optical tracking system. These 4 trajectories were then applied by the robot in the order that the corresponding procedures were applied to the porcine cadaver, and forces experienced by the spinal segment were recorded by the load cell. Starting from the initial neutral position as obtained from the intact cadaver, passive lumbar movements were replicated first, followed by the SMT. Each applied trajectory was separated by a 2 minute recovery time with 3 pre-conditioning trials executed prior to testing and data collection^13^.

### Serial dissection

Following application of all robotic trajectories in the intact specimen, spinal structures were then removed and/or transected and the same robotic trajectories repeated. In this way, the loading distribution within specific spinal tissues was quantified. Based on the findings reported by Funabashi *et al*.^24^, the following spinal structures were removed/transected (via scalpel unless otherwise noted) in the same order for all specimens: 1) supraspinous and interspinous ligaments (SL), 2) bilateral facet capsules, posterior facet joints (via rongeur) and ligamentum flavum (posterior joints, PJ), 3) intervertebral disc and anterior and posterior longitudinal ligaments (IVD).

### Data Analysis

For the SMT application, pressure data was plotted against time for each porcine cadaver by using the software provided by the manufacturer (Chameleon Visualization and Data Acquisition Software 2012, Version 1.7.0.6, Pressure Profile Systems. Inc., Los Angeles, CA). Maximum applied force and time to peak were extracted by the software and used for SMT force-time characterization.

For robotic kinematic replication, the resulting forces of each specimen were plotted against time. Peak and mean forces along each axis were identified by customized software (LabVIEW, National Instruments, Austin, TX). Peak force was considered to be the maximum measured force during the entirety of each applied trajectory. Mean forces corresponded to the average value of forces involving both the loading and unloading phase of each passive movement as well as preload and thrust phases of SMT. Using the same axis definitions as for the forces (see *Robotic Testing*), the values of L4 rotations relative to L3 where peak loads occurred were taken from the rotations of the robotic platform for each of the 4 trajectories.

Given that all passive lumbar movements and SMT application were performed in all specimens, each observation of forces generated during SMT and passive lumbar movements was considered to be a repeated measure. Although the data was not distributed normally, a repeated measures multivariate analysis of variance (MANOVA) was performed due to its robustness to non-normal data^25^ followed by a Bonferroni post-hoc analysis for pairwise comparisons using IBM SPSS Statistics for Windows, Version 22.0 (Armonk, NY: IBM Corp.). Statistical significance was set at an alpha value of 0.05.

## Results

### Spinal manipulative therapy characteristics

From pressure system recordings, SMT provided an average peak force magnitude of 524 N (SD: ±41 N), with a time to peak of 220 ms (±15 ms). This resulted in an average loading rate of 2.38 N/ms).

### Vertebral rotations

Rotations of L4 vertebrae relative to L3 at peak loads are shown in Table 1, stratified by the applied motion and the axis of rotation. These rotations are represented as Cardan angles in the rotation sequence of X-Y-Z about the fixed axes. In general, rotations caused by SMT were significantly greater compared to passive motions. This was specifically the case for passive flexion where the rotation about each axis was not only significantly different from SMT, but opposite in direction (with the exception of rotation around z-axis, which had equal direction to SMT). Given that flexion and extension are opposite motions, this finding was expected; that is, SMT could not create similar motion as both flexion and extension.

### Intact Specimen - Forces

**Figure 6:**
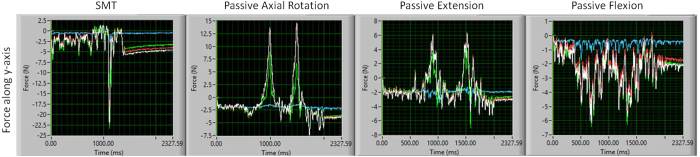
Representative example of force-time plots of raw forces experienced along the y-axis by the spinal segment during SMT and passive movements (left axial rotation, extension and flexion). White line represents the loads experienced by the intact specimen; red line represents the loads experienced by the whole segment after supra- and interspinous were removed; green line after bilateral facet joints, capsules and ligamentum flavum were removed and blue line after intervertebral disc, anterior and posterior ligaments were removed.

**Figure 7:**
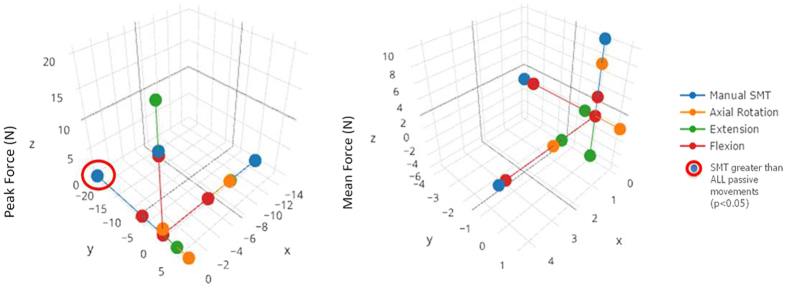
Average peak force and mean force experienced by the intact specimen during the application of SMT and passive lumbar movements of flexion, extension and left axial rotation. Data points circled in red indicate where SMT forces were significantly greater compared to all passive movements.

For general orientation, Fig. 6 shows a representative example of the raw forces (along y-axis) created by SMT and passive motions in a single, intact L3/L4 segment. Providing more detail, Table 2 presents the peak and mean forces for all specimens during SMT application and passive lumbar movements. Table 2 additionally displays statistical comparisons between SMT and passive movements of which there were 4 comparisons that were statistically significant, with all 4 having greater specific forces created during SMT. Among these 4 comparisons, 3 were notable in that the peak force created by SMT was in the anterioposterior direction and was significantly greater compared to the peak force generated by any of the passive lumbar movements (Fig. 7).

### Serial Dissection - Loading Distribution

**Figure 8:**
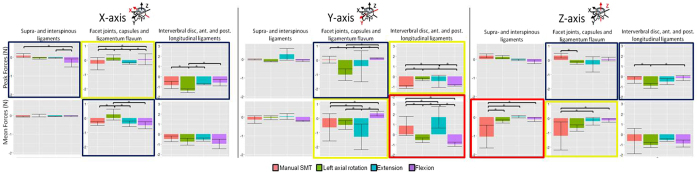
Average peak force and mean force experienced by specific spinal structures during the application of SMT and passive lumbar movements of flexion, extension and left axial rotation. Each section corresponds to a particular axis of movement (x-, y-, and z-axis). Each row within an individual section displays outcome variables (peak and mean forces) experienced by supra- and interspinous ligaments, bilateral facet joints, capsules and ligamentum flavum and intervertebral disc, anterior and posterior longitudinal ligaments (indicated on top of each column). Red boxes indicate significant differences between SMT and all three passive motions. Yellow boxes indicate significant differences between SMT and 2 passive motions. Navy boxes indicate significant differences between SMT and 1 passive motion (*p < 0.05).

Given that the objective of this study was to compare SMT to passive lumbar movements, we will not comment on results showing significant differences between the 3 passive movements; however, these results are displayed in Fig. 8.

Our serial dissection results demonstrated that although forces generated by SMT were not always significantly different from passive motions in the intact specimen, SMT sometimes created significantly different forces in specific tissues. In other words, the intact specimen may experience forces from SMT that are not significantly greater than passive movements, but in that same axis, specific spinal tissues may experience greater forces from SMT compared to one or more passive motions.

Overall, SMT created forces measured in distinct spinal tissues that were greater than those created by each of the passive motions. Specifically, SMT created significantly different forces in comparison to all passive motions in 2 cases (red boxes, 6 comparisons, Fig. 8). Of note, compared to all passive movements, SMT created significantly greater mean forces in the SL structures in the superioinferior direction.

Among these comparisons, some are of note in that SMT created significantly different forces in both specific tissues and the intact specimen. Specifically, SMT caused significantly greater peak anterioposterior forces in comparison to all three passive motions in the intact specimen while in specific tissues, SMT created significantly greater forces than passive flexion and axial rotation in the IVD structures and significantly smaller forces than passive extension in the IVD structures (Fig. 8).

Additionally, SMT also caused significantly different tissue loading than (i) two of three passive motions (yellow boxes, 8 comparisons, Fig. 8) and (ii) one of three passive motions (navy boxes, 6 comparisons, Fig. 8).

## Discussion

This study aimed to quantify and compare the forces experienced by spinal segments of a porcine cadaveric model during the application of SMT and passive lumbar movements. Our results support our stated hypothesis; SMT created unique loads when compared to passive lumbar movements of flexion, extension and left axial rotation. Interestingly, SMT generated loads within one specific axis (anteroposterior) that were greater than those created in the same axis by any of the three passive motions tested. This axis, the anteroposterior axis, was the direction in which the SMT was applied. In addition, SMT also created loading conditions that in some cases were significantly increased in the intact specimen as well as in individual spinal tissues (IVD structures). In other cases, SMT loading conditions were no different than passive motions in the intact specimen, but significantly different for a distinct spinal tissue. Although prior studies have investigated loading created during lumbar movement^16^,^26^ and SMT application^13^, this is the first study to quantify and compare the loads arising from both passive lumbar movement and SMT in a unified study with comparable specimens and testing protocol. Given that SMT appears to provide loading environments which are different from passive movements, it may be these unique SMT loads that confer its various effects, beneficial or otherwise.

SMT force-time characteristics measured here were found to be similar to those reported by previous investigations^27^,^28^. Likewise, the time for SMT application to reach peak force as well as the SMT loading rate are also in agreement with previous research^29^,^30^,^31^. These comparisons suggest that SMT applied in this experiment was representative of SMT applications performed on human subjects.

In addition, the relative vertebral motions observed during SMT in this current study (Table 1) are similar to those of our prior study^13^ which used a similar animal model. In both studies, the greatest relative rotation was observed around the z-axis (torsion). With respect to vertebral motions measured by other groups, Gal *et al*.^32^ observed significant relative flexion extension rotations (0.2–1.8°) during SMT application in unembalmed human cadavers. These comparisons suggest that, like the SMT application parameters measured in this study, the resulting vertebral kinematics arising from SMT are similar between porcine and human cadavers^33^,^34^. Importantly, such comparisons should be made with caution as coordinate systems between studies may differ. Given these cautions, relative vertebral rotations observed during both SMT and passive lumbar movements (Table 1) generally remained within ranges reported in the literature^35^,^36^,^37^,^38^,^39^,^40^,^41^,^42^,^43^.

As unique SMT loads occurred in both the intact segment as well as in specific tissues, it is possible that the physiological effects of SMT are tissue specific. Indirect support for this idea comes from prior studies showing that SMT creates unique strain patterns in facet joints compared to physiological axial rotation^44^, and that specific tissue properties change in those who are SMT responders^2^,^3^,^4^. Similarly, particular spines, spinal conditions, or tissues (e.g., degenerative spine or tissues) may not be impacted by these same loads which may help to explain SMT non-responders or when adverse events occur following SMT. Future studies are planned to investigate how these specific loads may influence specific physical responses.

Of note, the approach used in this study not only permits identification of unique SMT loads compared to passive motions, but to also observe if loads created by SMT exceed known thresholds for tissue injury. Previous investigations using finite element models have shown that high loading rates increase the risk of spinal structures injury^45^,^46^,^47^ as a result of the viscoelastic behavior of spinal structures. Specifically, Wang *et al*.^46^ assessed slow (0.6 N/ms and 0.003°/ms) and fast loading rates (6.6 N/ms and 0.03°/ms) of combined compression and flexion movements and found that high loading rates increased intradiscal pressure as well as forces and moments experienced by spinal structures. Additionally, El-Rich *et al*.^45^ investigated spine flexion and extension using three loading rates (0.05°/ms, 0.5°/ms and 5°/ms) and found that spinal injuries, such as bone fracture and ligament failure, can occur in loading rates higher than 0.5°/ms. Further, Wagnac *et al*.^47^ applied flexion, extension, anterior and posterior shear at low (0.1 m/s) and high rates (1.0 m/s for compression and 4.0 m/s for flexion and extension) and observed that loading rate significantly influenced spinal injury site. In the current study, SMT was applied with loading and rotation rates (2.38 N/ms, 0.04 m/s and 0.014°/ms) which are near or below the lower loading rates used in the abovementioned studies.

Additionally, the estimated stiffness of the L3/L4 spinal segment arising from SMT application was also smaller in magnitude when compared to the magnitude of stiffness observed to occur immediately before spinal injury^48^,^49^,^50^,^51^. Specifically, our data allows us to estimate L3/L4 spinal stiffness during SMT to be 0.33 Nm/deg in extension and 1.51 Nm/deg in axial rotation; values that are smaller than the extension stiffness described by Garges *et al*.^49^ of 10 Nm/deg and torsion stiffness described by Bisschop *et al*.^50^ of 5.7 Nm/deg.

Despite the greater anterioposterior forces created by SMT when compared to passive lumbar motion, the magnitudes of forces experienced during SMT are considerably smaller than those reported in previous research that investigated porcine spinal structural failure at 800 N to 8700 N^52^,^53^,^54^. Although the comparison between anterioposterior forces between the current study and previous investigations is limited due to methodological differences, anterioposterior forces created by SMT did not exceed 40 N.

It is important to note that, although the loading rates, stiffness values and load magnitudes obtained in this study fall below injury thresholds reported previously, our data cannot be used to suggest SMT is safe and/or would be unlikely to cause injury as our experiment was not constructed to assess SMT safety. Indeed, although porcine lumbar spine models have been described to be suitable models to investigate the human spine^33^,^34^, anatomical and biomechanical differences exist which also include a lack of biologically active tissues in our model (i.e., muscle). Therefore, extrapolation of these results to human living subjects is limited. Additionally, given the results reported by Funabashi *et al*.^24^, our observations are specific to the order in which spinal structures were removed from the specimen; a different order of tissue removal may affect the loads recorded within each spinal tissue, but not the results obtained for the intact specimen. Further, to avoid potential confounding factors due to viscoelastic behaviour^55^,^56^, this study standardized the order of robotic kinematic replication: passive movements were replicated first followed by the SMT. In this way, potential experimental differences due to changes in water content were standardized among specimens. In addition, although the processes used to perform passive movements were not quantified for their reliability, increased variability in passive motion application would make it more likely that loads created by SMT were similar to those created by passive movements – the opposite of our findings. Finally, as SMT was provided by a single clinician in this study, these results cannot be generalized to other clinicians or to all SMT techniques.

## Additional Information

**How to cite this article**: Funabashi, M. *et al*. Tissue loading created during spinal manipulation in comparison to loading created by passive spinal movements. *Sci. Rep.* **6**, 38107; doi: 10.1038/srep38107 (2016).

**Publisher's note:** Springer Nature remains neutral with regard to jurisdictional claims in published maps and institutional affiliations.

## Figures and Tables

**Table 1:** L4 rotation (°) (SD) relative to L3 created in intact cadaveric specimens at peak loads during the application of SMT and passive movements.

Motion	Rotation (°)		
	X (flx ext)	Y (lat bending)	Z (axial rot)
SMT	3.06 (1.00)^*,#^	−0.65 (0.52)^^,#^	−1.71 (1.20)^^,#^
Axial Rotation	1.40 (0.74)	−0.44 (0.74)	−1.24 (0.58)
Extension	3.17 (0.78)	−0.07 (0.45)	−0.20 (0.27)
Flexion	−0.58 (0.39)	0.32 (0.16)	−0.19 (0.12)

SD = standard deviation; SMT = spinal manipulative therapy.

^*^Significant difference (p < 0.05) in comparison with axial rotation.

^^^Significant difference (p < 0.05) in comparison with extension.

^#^Significant difference (p < 0.05) in comparison with flexion.

**Table 2:** Average and 95% confidence intervals of peak and mean forces experienced by the intact spinal segment during SMT application and passive physiological movements of flexion, extension and left axial rotation.

Motion	Forces					
	Peak			Mean		
	X	Y	Z	X	Y	Z
	(lateral)	(ant post)	(sup inf)	(lateral)	(ant post)	(sup inf)
SMT	−14.92 N; 95% CI:[−5.06, −24.78]	−22.23 N; ^*,^,#^ 95% CI:[−29.45, −15.02]	13.84 N; 95% CI:[22.85, 4.84]	4.32 N; 95% CI:[−4.08, 12.73]	−4.06 N; 95% CI:[−6.95, −1.17]	10.01 N; ^**^**^ 95% CI:[3.17, 16.85]
Axial Rotation	−10.16 N; 95% CI:[−4.05, −16.26]	7.03 N; 95% CI:[3.40, 10.67]	1.13 N; 95% CI:[15.87, −13.59]	2.12 N; 95% CI:[−3.46, 7.71]	1.17 N; 95% CI:[−0.66, 3.00]	7.08 N; 95% CI:[−1.24, 15.39]
Extension	−10.29 N; 95% CI:[1.34, −21.93]	3.90 N; 95% CI:[1.82, 5.99]	20.42 N; 95% CI:[32.10, 8.74]	1.73 N; 95% CI:[−4.24, 7.70]	−0.52 N; 95% CI:[−2.12, 1.07]	−6.28 N; 95% CI:[−2.12, 1.07]
Flexion	−6.53 N; 95% CI:[0.75, −13.81]	−6.33 N; 95% CI:[−9.52, −3.14]	−13.19 N; 95% CI:[22.85, 3.53]	4.07 N; 95% CI:[−1.58, 9.73]	−3.47 N; 95% CI:[−5.75, −1.19]	2.73 N; 95% CI:[−5.42, 10.89]

SMT = spinal manipulative therapy; ant post = anterioposterior; sup inf = superioinferior; flx ext = flexion extension; lat bend = lateral bending; ax rot = axial rotation.

^*^Significant difference (p < 0.05) in comparison with axial rotation.

^^^Significant difference (p < 0.05) in comparison with extension.

^#^Significant difference (p < 0.05) in comparison with flexion.
